# Microbial Contamination and Antibiotic Resistance in Fresh Produce and Agro-Ecosystems in South Asia—A Systematic Review

**DOI:** 10.3390/microorganisms12112267

**Published:** 2024-11-08

**Authors:** Pachillu Kalpana, Sandul Yasobant, Deepak Saxena, Christiane Schreiber

**Affiliations:** 1 Center for Development Research (ZEF), University of Bonn, 53113 Bonn, Germany; pkalpana@uni-bonn.de; 2 Department of Pharmacy, Faculty of Mathematics and Natural Sciences, University of Bonn, 53113 Bonn, Germany; 3 School of Epidemiology & Public Health, Datta Meghe Institute of Higher Education and Research (DMIHER), Wardha 442107, Maharashtra, India; 4 Department of Public Health Science, Indian Institute of Public Health Gandhinagar (IIPHG), Gandhinagar 382042, Gujarat, India; 5 Centre for One Health Education, Research & Development (COHERD), Indian Institute of Public Health Gandhinagar (IIPHG), Gandhinagar 382042, Gujarat, India; 6 Global Health, Institute for Hygiene and Public Health (IHPH), University Hospital Bonn, 53127 Bonn, Germany; 7 GeoHealth Centre, Institute for Hygiene and Public Health (IHPH), University Hospital Bonn, 53127 Bonn, Germany; christiane.schreiber@ukbonn.de

**Keywords:** fresh produce, antibiotic-resistant bacteria, antibiotic-resistant genes, pathogen, soil, water, agriculture

## Abstract

Fresh produce prone to microbial contamination is a potential reservoir for antimicrobial-resistant bacteria (ARB) and antimicrobial resistance genes (ARGs), posing challenges to food safety and public health. This systematic review aims to comprehensively assess the prevalence of bacterial pathogens and the incidence of ARB/ARGs in fresh produce and agro-ecosystems across South Asia. Twenty-two relevant studies published between 2012 and 2022 from three major scientific databases and the grey literature were identified. The results revealed a wide occurrence of microbial contamination in various types of fresh produce across South Asia, with a predominance of *E. coli* (16/22), *Salmonella* spp. (13/22), *Staphylococcus* spp. (5/22), and *Klebsiella* spp. (4/22). The agro-ecosystem serves as a complex interface for microbial interactions; studies have reported the prevalence of *E. coli* (1/4), *Salmonella* spp. (1/4) and *Listeria monocytogenes* (1/4) in farm environment samples. A concerning prevalence of ARB has been reported, with resistance to multiple classes of antibiotics. The presence of ARGs in fresh produce underscores the potential for gene transfer and the emergence of resistant pathogens. To conclude, our review provides insights into the requirements of enhanced surveillance, collaborative efforts, implementation of good agricultural practices, and public awareness for food safety and safeguarding public health in the region.

## 1. Introduction

The novel discovery of antibiotics in the 19th century transformed human health by saving millions of lives by treating diseases caused by bacterial infections [1]. The threat to this achievement came afterward with the increasing antimicrobial resistance (AMR), where microorganisms attain the ability to fight against previously effective antimicrobial drugs. This phenomenon of resistance in microorganisms leads to the extended persevere of infection in the body and triggers a higher risk of spreading to others [2]. AMR is now ranked among one of the top ten global health threats faced by humanity [3]. It is held to be responsible for nearly 1.27 million deaths worldwide in 2019, and this trend will increase globally if no remedial actions are taken [4]. It is estimated that global human mortality could reach up to 10 million people annually, with a cumulative loss in the global economy accounting for nearly USD 100 trillion by 2050 [4].

AMR varies geographically, but the expectation that more consumption of antibiotics in high-resource settings would lead to a corresponding higher burden of AMR-caused mortality was mistaken. On the contrary, the highest rates of deaths were reported in sub-Saharan Africa and South Asia [5]. Overall, 76.8 deaths in 100,000 of the human population have been experienced in South Asia. However, the high AMR burdens represent roles played by the prevalence of resistance and the underlying critical infections, which are reported higher in this region [6]. The other drivers that contribute to the observed higher burden in the South Asia region include inadequate health care systems, high accessibility to over-the-counter drugs, low biosecurity, unsafe food systems, and poor sanitation and hygiene, which also interpose to high and unregulated use of antibiotics [7].

The benefits of antibiotics do not restrict their usage only to human health care but also extend to the livestock and agriculture sectors [8]. The initial attempts to address this continually growing issue of AMR have prioritized human health care systems following livestock production [9]. The environmental sector, which could be contaminated with antimicrobial-resistant microbes and can generate reservoirs caused by discharges from wastewater treatment plants, for instance, has been less explored [10]. In the agricultural sector comprising food animal production and crop production areas, the focus has been paid to the former due to the high probability of transmission of zoonotic pathogens to humans through animal-sourced food. The majority (73%) of total antimicrobial consumption is reportedly used in animals raised for food, which is anticipated to rise even more with the projected growth in demand for animal proteins and with the necessity for strengthening livestock production systems in low–middle-income countries [11]. The usage of and demand for antibiotics established the reflection to focus on animal-sourced food in the food chain, but reductions in antimicrobial use in this particular agriculture sector may have a limited impact on tackling the issue of AMR infections [12,13].

In contrast, AMR transmission to humans through soil, water, and plants has been paid very minute consideration [14,15]. Crops cultivated in this broader setting for human consumption and animal feed enable the possibility of a direct link between the resistant microbes existing in the reservoir of agro-environment, humans, and animals [16]. In the food crop value chain, pre-harvest and post-harvest factors can contribute to contaminating fresh produce with antimicrobial-resistant bacteria (ARB). ARB harboring antimicrobial resistant genes (ARG) can be introduced to the crop environment through the application of animal waste as manure or crop fertilizer, by the use of contaminated irrigation water, or by residues from antimicrobial drugs used against crop pests [16]. Additionally, in pre-harvest conditions, cross-contamination of fresh produce by equipment or value chain actors can occur during harvest, and processing, washing, or handling in markets within the post-harvest period can play a role in ARB transmission [17].

In 2023, the Food and Agriculture Organization (FAO) and the World Health Organization (WHO) initiated promoting fruit and vegetable intake in healthy diets. Following the recommendation, the growing demand for fresh fruits and vegetables has increased and accounted for nearly 30% of global production in the last few years [18]. This increment has been gradual, with many worldwide variations in exports and imports [19]. However, the production of fruits and vegetables in Asian countries has almost doubled; hence, the exports are increasing faster than in other countries [20]. Fruits and vegetables are essential parts of providing nutrition, minerals, and vitamins to make a balanced diet and can prevent chronic diseases, including several micronutrient deficiencies, especially in developing countries [21]. Taking into consideration that vegetables undergo minimal or no processing in order to preserve the taste and their nutrient contents prior to ingestion by humans, fresh fruits and vegetables might serve as a vehicle for the transmission of human pathogens, ARB, and the related genes [22]. Until recently, risk analyses of foodborne AMR have focused almost exclusively on animal-based foods [23]. Moreover, AMR surveillance programs do not generally sample plant-based food, constraining baseline data for quantitative microbial risk assessments.

Most countries constituting the South Asia region are densely populated and share the issue of faster-growing AMR than any other region in the world [24]. The susceptibility to AMR contamination due to the close integration of people living near their livestock and agricultural areas creates a convenient setting for AMR spread [11]. The contamination of irrigation water resources by agricultural and industrial wastewater, lack of sanitation and hygiene measures, and the dependency on antimicrobials to compensate for poor animal husbandry practices associated with underdeveloped surveillance systems contribute more towards the faster-growing AMR in South Asia than in any other region in the world [25]. In addition, poor regulation in the supply chains of antimicrobials and fewer barriers to over-the-counter purchase of antimicrobials for the farmers, combined with limited access to agricultural extension services to acquire knowledge of agronomic techniques and skills to improve productivity, food security, and livelihoods, facilitate opportunities for misapplication of antimicrobials [26]. For these reasons, crop production systems in South Asia may be an underestimated source of dissemination of resistant bacteria to humans in those regions and potentially other parts of the world via international food supply chains.

In this review, we employed a systematic approach to explore the studies on microbial contamination and broad-spectrum patterns of AMR characterized in fresh produce for human consumption with a particular interest in South Asia. The objective of this review was to consolidate the current state of knowledge and data available and summarize the bacterial pathogens, antibiotic-resistant bacteria (ARB), and antibiotic-resistant genes (ARG) already detected and reported in fresh produce and the related agro-environment.

## 2. Materials and Methods

This study used a systematic review to categorize the studies conducted to answer a specific research question: which bacteria, antibiotic-resistant bacteria (ARB), and antibiotic-resistant genes (ARGs) are known to be widespread in the fresh produce and the agro-environment of the South Asia region? A protocol was developed according to the specified research question, including inclusion/exclusion criteria, search engines, and research sources. The selection and reporting of the relevant studies were performed using the Preferred Reporting Items for Systematic Reviews and Meta-Analyses (PRISMA) selection method [27]. The review protocol has not been registered. No automation tool was used in any step of this review process.

### 2.1. Information Sources

The three most important electronic scientific bibliographic databases, PubMed, Web of Science, and Scopus, were searched systematically for published peer-reviewed literature. Additionally, the grey literature was searched online via Google Scholar and Google. To ensure a comprehensive evaluation of the relevant literature, the reference lists of articles found by search and included in PRISMA analyses were scanned for all relevant published studies.

### 2.2. Search Strategy

Relevant published scientific studies were searched using the following series of steps: Initially, a preliminary search was performed to identify and gather keywords from the published literature, Google Scholar, and Google for each concept on the topic of interest until November 2023. The specified keywords were then used for the advanced search in PubMed, and the foremost step was to search the keywords independently to hit upon the MeSH terms in the MeSH hierarchy tree. The key search terms were divided into three domains: (i) antimicrobial resistance; (ii) agriculture, agriculture products, and farm produce; and (iii) study site/country. Boolean logic (“AND” and “OR”) was applied to combine the MeSH terms together for the search of published literature. The search was framed between the years 2012 and 2022. The search terms used to explore all three scientific databases with the Boolean operators for this study are shown in Table 1.

### 2.3. Study Selection Procedure

According to the PRISMA guidelines, the four steps adhered to the selection process in this review were (i) identification, (ii) screening, (iii) eligibility, and (iv) inclusion. The studies found by the database search were exported to a Microsoft Excel spreadsheet, where the first step was to eliminate duplicate entries by considering the title, authors, and publication year. The shortlisted studies obtained after the elimination step were further reviewed by independently screening their titles and abstracts by two authors. A study was considered potentially relevant if it mentioned antimicrobial resistance in combination with agriculture, agriculture products, or farm products-related terms. The set of studies consolidated after this screening was then subjected to a full article review by two authors individually for further evaluation. In order to evaluate only original studies, articles published in the form of review articles, books, book chapters, conference abstracts/proceedings, or guidelines were not considered, thus preventing information from being duplicated in the results. Next, pre-specified criteria for inclusion and exclusion mentioned in Table 2 were applied to determine which studies were relevant and should be finally evaluated. Only studies that demonstrated an explicit and clear link to the objective of the study and the review concept and those meeting all the pre-defined inclusion criteria were eligible to be included in the final synthesis.

### 2.4. Synthesis

The studies that met the set of inclusion criteria developed for the review were used to extract the relevant data, which were administered and merged in a data extraction spreadsheet created on Microsoft Excel. The data collection formats developed by Joana Briggs Institute (JBI) suitable for systematic review were employed for the data extraction process [28]. The author’s name, year of publication, study settings/location, sample characteristics, and outcome measures were collected. The heterogeneous nature of the studies found in the form of study design, type of samples, different methods, and breakpoints used for the antimicrobial susceptibility led to performing a narrative synthesis to synthesize the findings of the studies. Afterward, the studies were further scanned, and the data were extracted on the following factors: type of samples investigated, number of samples screened, type of microorganism reported, number of isolates found, the method used for antimicrobial susceptibility testing, resistance reported against antibiotics, and the specific AMR genes if reported. The data were then grouped based on the critical results found through narrative analysis of all the studies included in this review.

## 3. Results

### 3.1. Search and Study Selection

**Figure 1:**
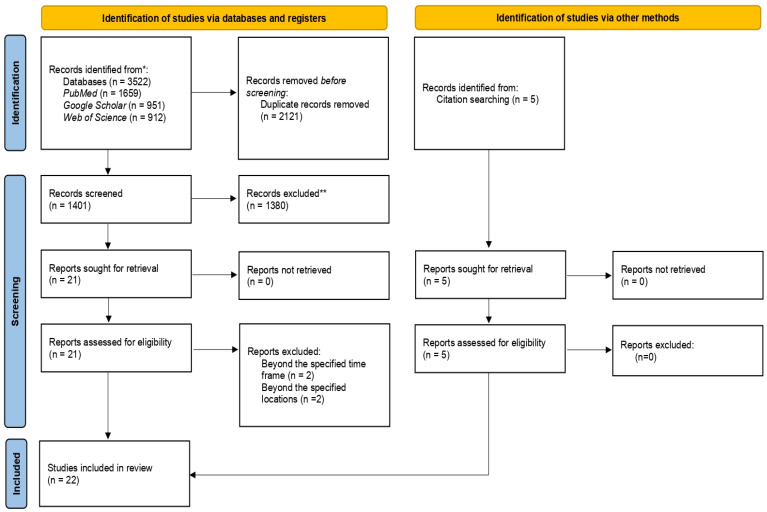
PRISMA flowchart diagram illustrating the selection process of studies for the systematic review. * Consider, if feasible to do so, reporting the number of records identified from each database or register searched (rather than the total number across all databases/registers). ** If automation tools were used, indicate how many records were excluded by a human and how many were excluded by automation tools.

The preliminary literature search throughout the electronic databases and the grey literature yielded after the duplication removal brought into 1401 studies. The titles and abstracts of all the studies were screened, and 1380 studies were eliminated in the first screening criteria due to the unrelated study concept. Finally, 26 studies, including five studies identified through hand search, were forwarded for full-text evaluation against the eligibility criteria, and 22 of them passed through eligibility assessment and were included in the final data synthesis. The complete study selection process followed for the review is picturized through the PRISMA flow chart in Figure 1.

### 3.2. Study Characteristics

**Figure 2:**
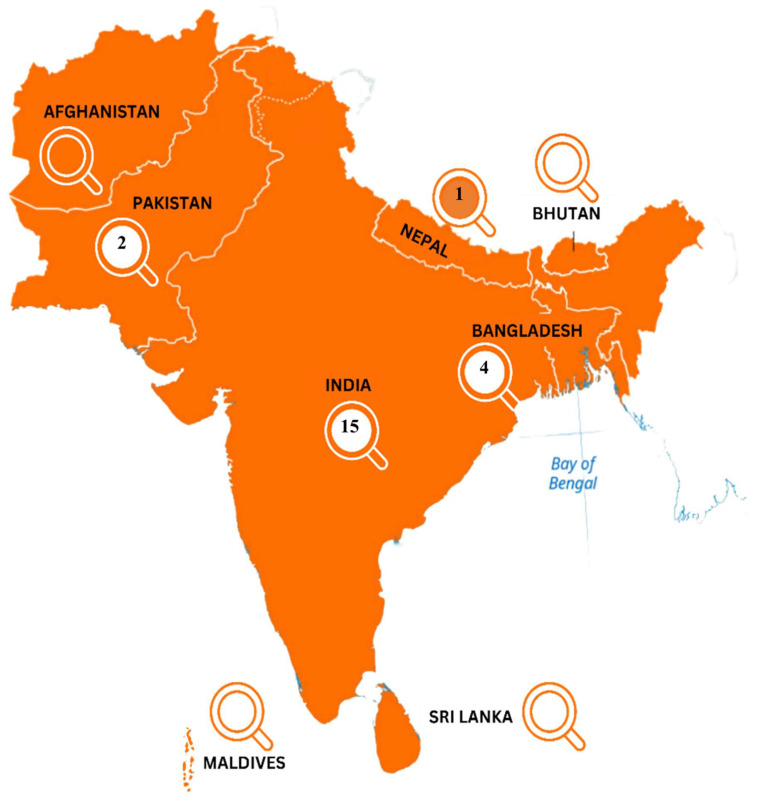
A pictorial illustration of the studies conducted in different countries of the South Asia region.

A total of 22 published studies were included in the final review process, engaging a varied number of samples, ranging from fresh produce to environmental samples representing the agro-environment. The focus area of this study, which is the South Asia region, comprises eight countries. These eight countries include Bangladesh, Bhutan, India, Maldives, Nepal, Afghanistan, Pakistan, and Sri Lanka. However, the distribution of the published articles in these countries varies greatly, with a range of 15 in one and none in the other part of this region. The highest number of studies conducted in the region was in India (n = 15), followed by Bangladesh (n = 4), Pakistan (n = 2) and Nepal (n = 1). No studies were reported from the remaining four countries that are part of South Asia. The country wise studies included in the review are illustrated pictorially in Figure 2. Table 3 represents the basic characteristics of all the included articles.

The observational studies were included in this review to provide data from the natural environment. In the included studies, researchers surveyed samples from the agro-ecosystem for the presence of microbial contamination and, in limited, also explored the AMR pattern of the microorganisms and reported the prevalence of antibiotic-resistant strains from the agro-ecosystem. However, only one study included the investigation of ARGs on vegetables and environment samples.

### 3.3. Sample Characteristics

#### 3.3.1. Setting of Reported Samples

**Figure 3:**
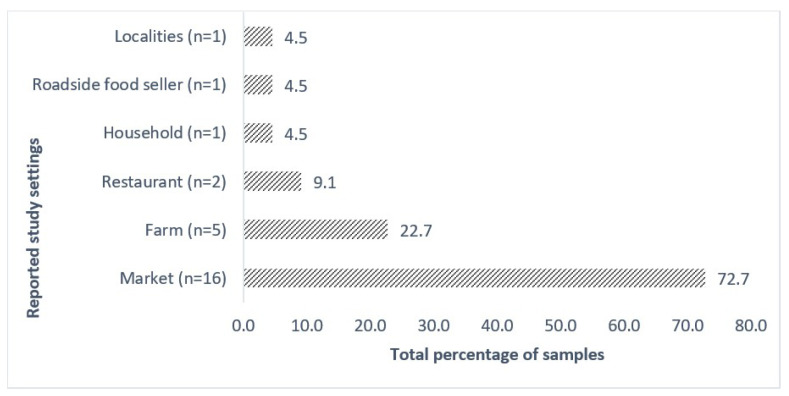
Distribution of reported sample settings of the publications.

Most studies collected and analyzed multiple sample types with a significant variance in the number of samples in the 22 included articles in this review. The majority of studies reported vegetables and fruits sampled from different categories of the market (72.7%, n = 16), which were stated as markets, local markets, retail markets, shops, wholesale markets, supermarkets, and food marts in the included studies. The studies reported from farms marked second in place (22.7%, n = 5) consisted of fresh produce along with related environment samples, as shown in Figure 3. The farms subset was considered for the studies that collected samples from agricultural fields, dairy farms, and grazing sites. The studies focused on ready-to-eat salads collected samples from hotels or restaurants (9.1%, n = 2). A study reported that samples were collected from roadside food stalls (4.5%, n = 1), which is very common in the geographical locations of the studies selected for this review. Interestingly, apart from the sites mentioned above, a study reported sample collection from households (4.5%, n = 1). On one side, from all these specific sample collection settings in the mentioned studies, one of the studies mentioned the sample collection from different localities (4.5%). The random sampling method was the most often reported method for sample collection in this final set of studies.

#### 3.3.2. Category of Samples

A significant percentage of reported samples focused on vegetables consumed in raw form by humans. Some of the studies’ raw consumed vegetables were also reported as salad vegetables. The group of vegetables sampled differs between the studies in terms of both the number and the type of vegetables analyzed. The more detailed review process revealed that the 22 studies reported different kinds of vegetables categorized into six groups for analysis purposes. The six groups consisted of fruits (tomato, cucumber, pepper, brinjal, chili, luffa, apple, papaya, lemon, mango, cantaloupe, pear, grapes, and chappan kaddu), root vegetables (carrot, radish, turnip, beetroot, onion, garlic, ginger, and potato), leafy vegetable (lettuce, fenugreek, and spinach), vegetable (cabbage, cauliflower, and broccoli), herbs (coriander and mint) and legumes (peas, cowpea, bean). The distribution is detailed in the Supplementary Materials.

However, the most frequently observed sample among all the studies was tomato (n = 13), followed by cucumber (n = 11), carrot (n = 10), cabbage (n = 9), and spinach (n = 8). The two studies that reported ready-to-eat salads or vegetable salads had a precise categorization in the type of sample analyzed and were similarly considered and analyzed for our review [30,44]. A few studies reported fruit samples, including apples, papaya, lemon, pear, and grapes [32]. Four studies involving the environmental samples alongside vegetables included farm soil, manure fertilizer, and irrigation water [31,43,48,49]. The detailed information on the samples in all the included studies is listed in Supplementary Table S1.

### 3.4. Microbial Contamination

In Table 4, the studies that have reported the contamination of samples by any microorganisms are also reported. The studies followed different methodologies as per the aim of the respective study to isolate and identify the type of microorganism. The studies focusing on microbial contamination also looked for the total bacterial counts along with any specific pathogenic microorganisms. The analysis of the methodologies followed by the studies shows that most of the samples were analyzed by microbiological culturing methods (18/22) to report the contamination of specific microorganisms or the total bacterial contamination. Three studies reported the contamination of samples using molecular techniques [35,37,48]. One of the studies did not report the contamination of particular microorganisms but reported the antibiotic-resistant bacteria [49]. The detailed methodologies, including the media, incubation time, and temperature, are summarized in the Supplementary Table S1.

#### 3.4.1. Microorganism Investigated

Contemplating the data obtained from the detailed study of the articles, the most frequently recorded species of interest were *E. coli*, *Salmonella* spp., *Staphylococcus* spp., and *Klebsiella* spp. The observations from these species comprised primary culture-positive sample records in vegetables and the soil, manure, and irrigation water samples. Other microorganisms reported in the study set included *Bacillus cereus*, *Listeria* spp., *Pseudomonas* spp. *Enterobacter* spp., *Vibrio* spp. *Proteus* spp., *Citrobacter* spp., *Serratia* spp., *Shigella* spp. *Acinetobacter* spp. across all sample groups. Many of the studies also deliberated on specific pathogens such as *E. coliO157:H7* and Diarrheagenic *E. coli* Pathotypes (DEPs). Two of the studies also reported scarce data on the yeast and fungal contamination of the vegetables during the search for this review.

#### 3.4.2. Microbial Contamination of Vegetable and Food Samples

All 22 articles investigated and successfully detected bacteria in the samples analyzed in the respective studies. The studies detected bacteria of more than 20 different genera in the group of vegetables and fruits, as represented in Table 4. Three of the studies detected the total number of bacterial counts for reporting the microbial load on the samples [29,34,38]. Five studies have reported the contamination as the number of bacterial isolates recovered in all the samples of the respective studies [32,34,36,44,47]. Among the prevalence of bacteria selected explicitly in most of the studies and also frequently observed bacteria were *E. coli*, found in 16 out of 22 included articles.

*E. coli* strains were mainly reported from cucumbers, tomatoes, carrots, cabbage, and leafy green vegetables. In one of the recent studies conducted in India, which reports among 830 samples including cabbage, fenugreek, coriander, spinach, and peppermint, 117 samples (14%) were contaminated with *E. coli* strains, and approximately 43% of them were identified as diarrheagenic *E. coli* pathotypes (DEPs). *Staphylococcus* spp. was reported in carrot, cucumber, onion, and tomato samples. In the studies that reported the contamination rate in all samples collected, the contamination range was found to be between 15% and 28.6%. Among the five articles that focused on and analyzed *Klebsiella* spp., the range of contamination was reported to be from 41% to 43%. The most common contaminated sample was reported to be tomato. The only study that reported the collection of vegetable samples at the household level in India also reported the presence of *Klebsiella* spp. *Staphylococcus* spp. and *Klebsiella* spp. were analyzed in six and five studies, respectively.

Some other microorganisms were also investigated and reported in the studies involving *Enterobacter* spp. (5/22), *Pseudomonas* spp. (4/22), *Listeria* spp. (3/22), *Bacillus* spp. (2/22), *Exiguobacterium* spp. (2/22), *Acinetobacter* spp. (3/22), and *Vibrio* spp. (1/22). The included studies also analyzed microbial contamination in different aggregated forms and reported as total viable counts, total heterotrophic count, total aerobic count, aerobic mesophilic count, psychotropic count, total Bacteroides, the total coliform counts, and total endophytic counts on the samples.

#### 3.4.3. Microbial Contamination of Agro-Ecosystem Samples

A study conducted in Bangladesh tested farm environment samples comprising manure, irrigation water, and soil and reported the presence of *E. coli* and *Salmonella* spp. in all kinds of samples. The most contaminated sample reported in this study was soil, with more than 90% of samples contaminated with *E. coli* and 70% with *Salmonella* spp., followed by manure with 80% of *E. coli* and 70% with *Salmonella* spp. The irrigation water was the least contaminated, with 45% of the sample positive for *E. coli* and 30% for *Salmonella* spp. Another study that tested rhizospheric soil of the vegetable samples reported detection of *Listeria monocytogenes* in ten of the 200 samples collected in India. The other two studies that tested the soil and manure samples have not mainly focused on the detection of any specific bacteria. A study focused on soil samples using next-generation sequencing (NGS) technology to analyze the whole bacterial community present in the soil sample collected from different agricultural fields and concentrate on reporting the total bacterial count, *Proteobacteria*, *Acidobacteria*, *Actinobacteria,* and *Chloroflexi*.

### 3.5. Antimicrobial Susceptibility Testing

In 18 of the 22 studies, data on the antimicrobial resistance patterns of the isolated microorganisms were reported. Two primary methods have been reported in the studies for detecting the AMR patterns of the reported microorganisms. In 13 studies, the Kirby Bauer method of the disk diffusion test was employed, and in 2 other studies, the broth microdilution method was used for AMR detection by CLSI standards. In all the studies that followed the disk diffusion method, Muller–Hinton Agar was used, and the plates were incubated for 24 h at 37 °C after bacteria spread and antibiotic disc placement. In the other three studies, one study did not mention the method used to identify the AMR pattern. The other study calculated the percentage of ampicillin-resistant bacteria only by plating the diluted soil samples directly on an LB agar medium containing 100 µg/mL ampicillin. The study that profiled antibiotic resistance of manure used the serially diluted sample, followed by plating on a nutrient agar medium containing fluconazole as an antifungal (50 mg L^−1^) agent. The eight selected antibiotic discs were then placed on plates and incubated for 2 days at 30 °C.

Fifteen out of the eighteen studies followed the interpretive criteria and standards of Clinical and Laboratory Standards Institute (CLSI) guidelines for reporting the resistance profile of the isolated microorganisms. The details of the studies that reported AMR patterns of the microorganism tested, including the antibiotics tested and the resistance reported, are tabularized in Supplementary Table S1. The three other studies that were reported followed a slightly different approach, as mentioned above, for reporting AMR patterns, which included not the isolates but the direct samples that were tested in these studies.

#### 3.5.1. Antimicrobial Susceptibility Pattern

A significant difference was observed in the number of antimicrobial drugs applied to analyze the resistance and susceptibility patterns of the same type of microorganisms. The number ranges from 5 to 23, found in different studies. Due to the number and type of tested antimicrobials, the resistance patterns of all the isolates were diverse among the studies. The details regarding the AMR susceptibility pattern reported in the included studies are summarized in Supplementary Table S1.

#### 3.5.2. Extended Spectrum Beta-Lactamase (ESBL)

The three articles identified positive *E. coli* isolates and used a double-disc diffusion method to categorize them further as ESBL and non-ESBL producers. The highest percentage of ESBL producers (62%) was reported in the total number of Gram-negative isolates from vegetable salads. The 23 *E. coli* isolates identified from fruit, root vegetables, leafy vegetables, and vegetable samples reported only two isolates as ESBL producers. The ready-to-eat salads analyzed in one of the studies reported a total of nine ESBL producers, which included four *E. coli* and five *Salmonella* spp.

#### 3.5.3. Other Specific Resistance Bacteria

One of the included studies focused on some particular resistance patterns among the *E. coli* isolated from the vegetable and fruit samples. This specific study reported 8 ESBL-producing isolates from the 141 *Enterobacteriaceae* isolates and further identified 22 Amp-C-β producers, 6 carbapenem resistance, and 6 metallo-β-lactamase-producers.

### 3.6. ARG in the Fresh Produce and Agro-Ecosystem

Although molecular methods were employed in some studies to identify the virulent genes and evaluate the related analysis, there was only one study found in our review of 22 papers that were engrossed in reporting the prevalence of ARGs in fresh produce and the agro-ecosystem samples collected in this particular study and the details are shown in Table 5. This study was conducted in Bangladesh and focused on tetracycline resistance genes, particularly *tetA* and *tetB*, the *ESBL* gene *SHV*, and the erythromycin-resistant gene *ereA*.

## 4. Discussion

The included studies in this review reported the microbial contamination of fresh produce in diverse South Asian settings. While 22 articles are included in this more detailed review, 16 studies explore the reporting of resistant strains of human pathogenic microbes isolated from fresh produce and/or environment samples. We employed a systematic approach to extract the data from the included studies about the significant microorganisms reported on the fresh produce and the related environment samples. In the case of characterizing AMR in focused samples, both the phenotypic and genotypic results are analyzed. However, a firm conclusion could not be made on the prevalence of the microorganism and the relative importance of different kinds of resistance and AMR patterns because of the substantial heterogeneity between study methods and conditions; nonetheless, some broad, indicative patterns emerge from our analysis.

### 4.1. Relevant Vegetables and Fruits Within Reviewed Studies

Among the 22 studies that were included in the more detailed review process, around six different groups of fresh produce, including root vegetables, leafy vegetables, vegetables, herbs, fruits, and legumes, were studied. Some studies included fruits or samples from ready-to-eat salads comprising most of the sample types mentioned in the group. Among all the fresh produce samples listed in the results, cucumber, tomato, and carrot were found to be the predominately sampled due to their high and routine consumption in raw form and also a significant contribution to salads [32,35,36,37,38,42,44]. Studies from the South Asia region considered sampling majorly from different levels within the distribution chain of fresh produce. However, the most significant sampling was conducted from markets, mainly local, retail, and supermarkets, and it was rarely reported from street vendors. Some of the studies also sampled from the farm environment, including soil, irrigation water, and manure [31,43,48,49]. The apparent reason behind the selection of the location considered for sampling is unclear. Still, it might be because the markets are easy to access and provide a wide variety of samples at the same place, which is the main point within the farm-to-fork supply chain where it reaches the consumers.

### 4.2. Relevant Bacteria of Fresh Produce Within the Reviewed Studies

Among the prevalence of bacteria reported explicitly in most of the studies, *E. coli* was found in the highest numbers in 16 out of 22 included articles. Therefore, *E. coli* acts as the most popular fecal indicator bacterium in scientific research on food contamination in the South Asian region. This is in line with WHO guidelines with surveillance strategies concerning food hygiene and also with scientific research in other parts of the world due to the species’ characteristics multiplying only within their specific ecological niche, which is the intestine of humans and animals, but not within the environment [15]. *E. coli* strains were mainly reported from cucumbers, tomatoes, carrots, cabbage, and leafy green vegetables. In one of the recent studies conducted in India, which reports among 830 samples of leafy vegetables, including cabbage, fenugreek, coriander, spinach, and peppermint, 117 samples (14%) were contaminated with *E. coli* strains, and approximately 43% of them were identified as diarrheagenic *E. coli* pathotypes (DEPs) [50]. The DEP strains were also reported in leafy and non-leafy vegetables, including cucumber, carrot, and salad samples analyzed in another study in Pakistan [33]. That underlines the function of *E. coli* not only as an indicator but as a relevant pathogen within the South Asian Region, especially India and Pakistan. Among all pathogens in fresh produce, *E. coli* was found to be the predominant one. This could be the result of its ubiquity in nature [43], which is also one of the reasons for the indicator suitability of the species. The methodical ease in detection and isolation of *E. coli* also primes this microorganism as one of the major selected and detected in studies.

The second-in-line of the most common bacteria tested was *Salmonella* spp. 13 out of 22 articles reported the analysis of *Salmonella* spp. on fresh produce. For example, a study from Bangladesh reported the presence of Salmonella spp. on the tested samples of lettuce, cucumber, tomato, and coriander leaf [42]. Another study in Bangladesh, where the samples were collected directly from the farm, reported a higher prevalence, with approximately 50% positive samples of all the vegetables [43]. In Nepal, *Salmonella* spp. was detected in ready-to-eat salads collected from hotels and restaurants [44]. Possible reasons for the high prevalence of *Salmonella* could include poor hygiene and improper handling at markets and restaurants [44]. The improper management of manure in the production environment of fresh produce may be the cause of transmission of *E. coli* and *Salmonella* spp. to vegetables [43]. Colonization and persistence on the plant surfaces with significantly increased virulence potential of both species are stated to aid the formation of reported specific phenotypic traits of biofilms along with the production of cellulose and curli-fimbriae [41].

The detection of *Staphylococcus* spp. and *Klebsiella* spp. was reported in five and four studies, respectively. *Staphylococcus* spp. were reported to have recovered from carrot, cucumber, onion, and tomato samples [30,32,33,37]. One of the two studies that reported contamination of fruits with *Staphylococcus* spp. were conducted in India, but the percentage of positive samples could not be calculated due to a lack of information about the total numbers investigated [32]. Among the four articles that detected the presence of *Klebsiella* spp., the most common contaminated sample was reported to be tomato. One of the studies that reported the collection of vegetable samples at the household level in India reported the presence of *Klebsiella* spp. [45].

Other microorganisms that were often investigated and detected on fresh produce and fruits were *Listeria* spp., *Pseudomonas* spp., *Vibrio* spp., *Bacillus* spp., and *Enterobacter* spp. All the mentioned bacteria genera are known to be human pathogens; however, some of them are conditionally pathogenic. A few of the articles also reported microbial contamination of the fresh produce in the form of total viable counts, total coliform counts, and total endophytic counts [33,36,37,42].

Fresh produce contaminated with all the bacterial pathogens creates a food safety issue that is associated with many different kinds of human infections. Therefore, surveillance programs, including plant-based food samples, are needed, and the reported foodborne pathogens should be included in the panel of already monitored bacterial pathogens.

### 4.3. Relevant Bacteria of Agro-Ecosystem Within the Reviewed Studies

The reviewed studies (n = 4) investigated farm environment samples comprising manure, irrigation water, and soil/rhizospheric soil. The reported presence of *E. coli* and *Salmonella* spp. in all kinds of environment samples with the highest contamination frequency of soil samples (90% positive for *E. coli* and 70% positive for *Salmonella* spp.) [43] underlined ubiquitous fecal contamination. *E. coli* is one of the most prevalent facultative anaerobic bacteria in the gastrointestinal tract of humans and animals. The general practice of applying cow dung as manure in many of the agricultural practices followed in the South Asia region could be the reason for the highest prevalence of the reported species in soil samples. The finding that manure was also frequent, but irrigation water was least contaminated [43] underlined the different importance of transmission routes.

The abundance of Listeria monocytogenes detection in the rhizospheric soil of vegetable samples (10/200 samples positive) indicates, in contrast, that the transmission of *Listeria* spp. by raw vegetables is a minor health threat in India [31]. However, that pathogen is generally stated to be ubiquitous in the environment.

#### Relevant Antibiotics, Resistance Levels, and Resistance Patterns Within the Reviewed Studies

Antibiotic resistances were not studied very often among the reviewed studies. However, the isolates’ resistance levels and patterns were diverse among the studies due to the number of tested species and antimicrobials. Besides the standard methods followed for the AMR susceptibility pattern, two studies that tested AMR in soil and manure samples reported AMR following a slightly different procedure. In both studies, the samples were tested instead of the isolated microorganisms. The samples were diluted and plated on agar plates.

In view of *E. coli* and *Salmonella* spp. from the vegetable samples, the highest resistance levels were reported to azithromycin (100%), followed by tetracycline and erythromycin, and found to be similar in the case of isolates from soil, manure, and water samples [43]. The antibiotic resistance levels and patterns exhibited by *Salmonella* spp. also differ according to a second study that confirms the antibiotics that are most commonly used in the Southeast Asia region are erythromycin, azithromycin, and tetracycline [36,51].

Concerning ESBL-producing pathogens, the prevalence was reported in vegetable salads, ready-to-eat salads, and fruits [30,34,44,47]. The results of a small study conducted in India that found both ESBL-producing *E. coli* and non-ESBL producers in similar abundance, on top of significant differences in multi-resistance levels between the groups (up to nearly double in ESBL bacteria), have to be questioned in view to certainty and generalizability of that results, which we rate to be low due to a small sample size (n = 6) [30]. A study of more certainty because of a bigger sample size detected a much lower ESBL abundance of 6% (8/141) in *Enterobacteriaceae* isolates. A similar abundance was shown for carbapenem resistance and metallo beta lactamase-producers of 6% each. A much higher abundance of 16% was identified for AmpC beta-lactamases producers [47]. *Listeria monocytogenes* antibiotic resistance profiles showed the highest resistance to ciprofloxacin and cefotaxime antimicrobials, similar to those isolated from vegetable and soil samples [31].

Research interest in multi-resistances of vegetable-associated bacteria within the South Asian region during the last decade, in general, seems not to be very high due to the small number of papers dealing with that topic. *E. coli* and *Salmonella* spp. isolates reported from fresh produce were resistant to multiple antibiotics of clinical importance, such as erythromycin [44]. In the case of *Klebsiella* spp., three major antibiotics that were reported to be resistant were Amoxycillin, Ceftriaxone, and Tetracycline [36]. Resistance patterns of the 75% isolates of *Listeria monocytogenes* from vegetables and 50% from rhizospheric soil samples exhibited resistance to ciprofloxacin and cefotaxime, underlining the reduction efficiency of the particular antibiotics [31].

Following the explanations by Saptoka et al. (2019), the enteric bacteria fecal flora is specified to be substantially resistant, and *E. coli* is considered the leading transporter of antimicrobial resistance [44]. The increasing amount of antibiotics in the livestock sector affects the entire agro-ecosystem. The soil of the farm is heavily contaminated with resistant bacteria out of manure, which may have contaminated the vegetables produced on the contaminated soil. The use of unsafe water for irrigation could also contribute to the spread of ARB on the vegetables and increase prevalence in the agro-ecosystem [44]. Still, it may be less relevant regarding low contamination levels found by Abdus Sobur et al. (2019) [43]. The unhygienic conditions at the markets and lack of personal hygiene are also associated with the contamination of fresh produce with ARB. The lack of knowledge and awareness among the food handlers in restaurants with the ready-to-eat salad preparation steps further increases the risk of spreading pathogens and ARB on the plate [44].

Only one article presented resistance findings at gene levels in *E. coli* and *Salmonella* spp. isolated from irrigation water, vegetables, soil, and manure samples. General abundance levels of the resistance genes investigated increase in this order. The four resistance genes *tetA*, *tetB* (both responsible for resistance against tetracycline), *SHV* (beta-lactamases and ESBL), and *ereA* (encoding for Erythromycin Esterase) selected for testing, and the fact that the tetracycline resistance genes were most prevalent [43], underline the importance of Tetracyclines and Erythromycin (or their loss in effectivity) within the South Asian region, and especially for Bangladesh, where the study was conducted and tetracycline is one of the widely used antibiotics [43]. But, other than in the phenotypical testings [43], no macrolide resistance was verified by ARG here. Thus, it could be supposed, but is not indicated in the studies and therefore has to be further verified, that beta-lactam antibiotics may be used more often than macrolides in the South Asian region, or/and ESBL-based resistance may be worse than macrolide resistance in clinical surveillance and therapy.

## 5. Limitations

Our review systematically evaluated the microbial contamination, the prevalence of ARB and ARGs in fresh produce, and the environment samples of the agro-ecosystem of the South Asia region. In the narrow sense, the articles analyzed precluded us from reporting the multiple routes and exposure pathways that might be involved in the contamination of fresh produce or identifying practices that could aid in mitigating these risks. Any risk assessment regarding the extent of the human health risks associated with the consumption of contaminated fresh produce containing pathogens, ARB, or ARGs, was not the aim of this review.

Most of the studies focused on the prevalence of specific bacterial pathogens on samples, which limited the data on the presence of other foodborne pathogens. It is not always clear if the selection of investigated and reported species and resistances in each study is based on medical evidence within the South Asian region, e.g., clinical surveillance and therapy or personal researcher preferences.

In studies that reported the detection of ARBs by aggregating the phenotypic results, the information on the total number of samples tested or provided full susceptibility was missing. The analysis of the collected data from the studies often reports only the presence of resistance instead of the prevalence. The publication bias related to the studies with the resistance results prioritized for publication, leading to conclusions of resistant organisms on fresh produce, was unavoidable. Studies reporting the presence of resistant microorganisms reported the prevalence among the isolates instead of the collected sample groups. The resistance profiles of the same bacteria were found to be diverse among the studies owing to location, number of samples, types of samples (vegetable, soil, water, and manure), choice of antibiotics investigated, and detection and identification procedures followed. Due to the finding of heterogeneity among the included articles, a statistical analysis to prove significance was not performed. The lack of studies focussing on detecting ARGs in samples limited the analysis.

Thus, the reviewed studies showed a broad variation in study designs and a lack of standardized methods and data reporting, which challenged the comparability and understanding in our review and impeded the identification of generalizable, clear, and significant facts, such as the situation or trend applicable to the South Asian region.

The review showed an urgent need to standardize detection methods for environmental samples. In our experience, the practice of existing standards optimized for clinical samples or such with low background flora cannot be used due to the high microbial background in environmental matrices like soil, plants, manure, etc.

## 6. Conclusions

This review contributes to understanding AMR occurring in the fresh produce and agro-ecosystem. Our results indicate that the presence of pathogenic microorganisms on fresh produce that harbor resistance traits reflected by the presence of ARB and ARGS pose potential public health risks for consumers. The most frequent produce type found included raw-consumed vegetables or salad vegetables consumed with minimal processing, including tomato, cucumber, carrot, cabbage, and spinach. The most commonly encountered bacterial isolates contaminating the food and food-producing environment include *E. coli*, *Salmonella* spp., *Staphylococcus* spp., and *Klebsiella* spp. The reported bacteria were resistant to the most commonly used antimicrobials by humans in the South Asia region, such as tetracycline and erythromycin in fresh produce and agro-ecosystems in the published literature. This represents the complexity of contamination pathways involving human and environmental sources. The results from this review indicate potential food security and food safety issues and significant AMR exposure risk for consumers. The data on antimicrobial-resistant bacteria in a wide range of plant-origin foods reported from various regions of the world indicate the possible route of dissemination of antimicrobial-resistant bacteria and their genes to humans [52]. This highlights the essential need to incorporate plant-based food in AMR-related surveillance systems. In addition, including samples from the immediate food production environment, such as soil, irrigation water, etc., is equally vital in complementing the AMR in food and environmental dimensions. However, the considerable challenge for incorporating plant-based food in surveillance systems is the lack of standardized indicators of microbes, methods of measuring resistance, and types of AMR, which are reported in this review. This challenge requires considerable input from various stakeholders, but it is necessary for systematic surveillance, reporting, and comparison of data worldwide to assess potential risks. Future studies should focus on the South Asia region to quantify ARB/ARGs in fresh produce and its production environment.

## Figures and Tables

**Table 1:** Search strategy for the systematic review.

**Key search terms**	**Antimicrobial resistance-related terms** **(Combined by ‘OR’) (i)**	**Agriculture, agriculture products, and farm produce-related terms** **(Combined by ‘OR’) (ii)**	**Study site/country** **(Combined by ‘OR’) (iii)**
	(“antibiotic*” OR “antimicrobial*” OR “antimicrobial resistan*” OR “antibiotic resistan*” OR “antibiotic resistant bacteria” OR “antibiotic resistant gene” OR “antimicrobial resistant organism” OR “antibiotic resistant pathogen”)	(“agriculture” OR “fresh produce” OR “vegetables” OR “salad” OR “fresh agriculture products” OR “raw vegetables” OR “salad vegetables” OR “fruits” OR “leafy green” OR “farming”)	(“india” OR “pakistan” OR “afghanistan” OR “bangladesh” OR “bhutan” OR “maldives” OR “nepal” OR “sri lanka” OR “south asia”)
Filters: from 2012 to 2022			

**Table 2:** Inclusion and exclusion criteria followed for the systematic review.

**Inclusion Criteria**	Studies that detect and/or quantify bacteria, ARB, and/or ARGs on agriculture products (plant/plant products/vegetables/leafy green/fruits), and/or in agriculture production environments (soil/manure/water)
	Studies that detect and/or quantify bacteria, ARB, and/or ARGs in mixed or ready-to-eat salads
	Studies conducted in South Asian countries
	Full-text-peer-reviewed journal articles and grey literature
	Language: English
**Exclusion Criteria**	Studies that did not include any fresh agriculture product
	Studies that include data on animal agriculture (e.g., dairy, meat, aquaculture, poultry) only
	Studies that include data on animal-based foods (e.g., egg, milk, beef, pork, chicken, etc.) only
	Studies that did not have data from the South Asian countries
	All types of review articles

**Table 3:** Basic characteristics of the included study articles.

Article No.	Authors	Publication Year	Country	Specific Localities	Frequency	Category of Locality							Category of Sample (Fresh Produce)						Category of Sample (Environment)			Studies Reported		
						Market	Farm	Restaurant	Hotel	Household	Roadside Food Seller	Other	Root Vegetable	Leafy Vegetable	Vegetable	Herbs	Fruit	Legumes	Soil	Water	Manure	Microorganism Detected	Antibiotic-Resistance Tested	Antibiotic-Resistant Genes
1	Chaturvedi, M., Kumar, V., Singh, D., and Kumar [29]	2013	India	Shops	8	X							X		X			X				X		
2	Rasheed, M. U., Thajuddin, N., Ahamed, P., Teklemariam, Z., and Jamil, K. [30]	2014	India	Localities	12							X	X	X	X		X					X	X	
3	Soni, D. K., Singh, M., Singh, D. V., and Dubey, S. K. [31]	2014	India	Agricultural Farm	N/A		X							X	X		X	X	X			X	X	
4	Mathur, A., Joshi, A., Harwani, D., and Ganga, M. [32]	2014	India	Market	N/A	X							X	X			X					X	X	
5	Kabir, A., Das, A. K., and Kabir, M. S. [33]	2015	Bangladesh	Local market, Super market	7	X							X		X		X	X				X	X	
6	Chellapandi, K., Ralte, L., Malsawmtluangi, L., Masih, L., Kumar Singh, K. T., and Boro, D. [34]	2015	India	Vegetable market	3	X							X		X		X					X	X	
7	Shah, M. S., Eppinger, M., Ahmed, S., Shah, A. A., Hameed, A., and Hasan, F. [35]	2015	Pakistan	Local vegetable market and restaurant	N/A	X		X					X	X			X					X	X	
8	Kundu, S. K., and Islam, M. T. [36]	2016	Bangladesh	Local market	N/A	X											X					X	X	
9	Nithya, A., and Babu, S. [37]	2017	India	Local market	N/A	X							X				X					X		
10	Mritunjay, S. K., and Kumar, V. [38]	2017	India	Local market, retail market, local producer	N/A	X							X	X	X	X	X					X		
11	Rabins, S. L., Bhattacharya, A., Ajay kumar, V. J., and Vijayan, C. [39]	2018	India	Vegetable market	N/A	X										X						X	X	
12	Kundu, A., Wuertz, S., and Smith, W. A. [40]	2018	India	Wholesale markets, foodmarts, roadside food seller	3 5 1	X					X					X	X					X		
13	Verma, P., Saharan, V. V., Nimesh, S., and Singh, A. P. [41]	2018	India	Local market, agricultural fields	70 40	X	X						X	X			X					X	X	
14	Ahmed, S., Siddique, M. A., Rahman, M., Bari, M. L., and Ferdousi, S. [42]	2019	Bangladesh	Chain shops, wholesale market, retail market	3 3 6	X								X		X	X					X	X	
15	Abdus Sobur, M., Al Momen Sabuj, A., Sarker, R., Taufiqur Rahman, A. M. M., Lutful Kabir, S. M., and Tanvir Rahman, M. [43]	2019	Bangladesh	Dairy farm	4		X							X			X		X	X	X	X	X	X
16	Sapkota, S., Adhikari, S., Khadka, S., Adhikari, M., Kandel, H., Pathak, S., Pandey, A., and Pandey, A. [44]	2019	Nepal	Restaurant and hotel	N/A			X	X				X				X					X	X	
17	Ghafur, A., Shankar, C., GnanaSoundari, P., Venkatesan, M., Mani, D., Thirunarayanan, M. A., and Veeraraghavan, B. [45]	2019	India	Shops household	14 8	X				X			N/A	N/A	N/A	N/A	N/A	N/A				X	X	
18	Ali, B. [46]	2019	Pakistan	Vegetable shop, vegetable market, superstore	N/A	X							X		X							X	X	
19	Saksena, R., Malik, M., and Gaind, R. [47]	2020	India	Retail vendor, wholesale market	N/A	X							X		X		X					X	X	
20	Rathore, P., Joy, S. S., Yadav, R., and Ramakrishna, W. [48]	2021	India	Agricultural field, non-agricultural field	3 1		X												X			X	X	
21	Shrivas, V. L., Choudhary, A. K., Hariprasad, P., and Sharma, S. [49,50]	2021	India	Farming site, grazing site	18 32		X														X		X	
22	Priyanka, Meena, P. R., Meghwanshi, K. K., Rana, A., and Singh, A. P. [50]	2021	India	Retail shops, vendors, agricultural markets, supermarkets	14 25 9 7	X								X	X	X						X	X	

N/A means not applicable

**Table 4:** Microbial contamination of fresh produce and agro-ecosystem in the included study articles.

Article No.	Publication Year	Country	Category of Locality	Frequency	Category of Sample	Samples Reported	Frequency	Microorganism Tested
1	2013	India	Market	8	Root vegetable	Potato	4	Total aerobic bacterial count
					Vegetable	Carrot	4	Yeast and mold count
					Legumes	Radish	4	*E. coli*
						Onion	4	Coliform count
						Garlic	4	
						Ginger	4	
						Cauliflower	4	
						Cabbage	4	
						Peas	4	
2	2014	India	Other	12	Fruit	Cucumber	30	*E. coli*
					Root vegetable	Tomato		
					Leafy vegetable	Beetroot		
					Vegetable	Radish		
						Carrot		
						Spinach		
						Cabbage		
						Lettuce		
3	2014	India	Farm	N/A	Fruit	Brinjal	20	*Listeria monocytogenes*
					Leafy vegetable	Chappan kaddu	20	
					Vegetable	Chilli	20	
					Legumes	Tomato	20	
					Environment	Spinach	20	
						Cauliflower	20	
						Cabbage	20	
						Broccoli	20	
						Dolichos-Bean	20	
						Cowpea	20	
						Soil	200	
4	2014	India	Market	N/A	Fruit	Mango	N/A	*Bacillus*
					Root vegetable	Lemon		*Lactobacillus*
					Leafy vegetable	Papaya		*Caynebacterium*
						Apple		*Streptococcus*
						Cucumber		*Staphylococcus*
						Chilli		*Micrococcus*
						Luffa		*Pseudomonas*
						Tomato		*Enterobacteriaceae*
						Potato		
						Onion		
						Spinach		
5	2015	Bangladesh	Market	7	Fruit	Tomato	2	Total heterotrophic bacteria
					Root vegetable	Carrot	2	Total coliform bacteria
					Vegetable	Radish	2	Fecal coliform
					Legumes	Turnip	2	*Staphylococcus aureus*
						Cauliflower	2	*Pseudomonas* spp.
						Cabbage	2	*Listeria* spp.
						Bean	2	*Salmonella* and *Shigella* spp.
								*Vibrio* spp.
								*E. coli*
6	2015	India	Market	3	Fruit	Tomato	27	Total microbial load
					Root vegetable	Potato		*E. coli*
					Vegetable	Cabbage		*Enterobacter* spp.
								*Klebsiella* spp.
								*Proteus* spp.
								*Citrobacter* spp.
								*Serratia* spp.
								*Staphylococcus* spp.
7	2015	Pakistan	Market Restaurant	N/A	Fruit	Cucumber	200	*E. coli*
					Root vegetable	Tomato		
					Leafy vegetable	Carrot		
					Vegetable	Spinach		
						Lettuce		
					Ready-to-eat salad		40	
8	2016	Bangladesh	Market	N/A	Fruit	Tomato	7	Total viable count
						Cucumber	7	Total coliform count
								Total staphylococcal count
								*Klebsiella* spp.
								*Enterobacter* spp.
								*Shigella* spp.
								*Citrobacter* spp.
								*E. coli*
9	2017	India	Market	N/A	Fruit	Cucumber	N/A	Density endophytic bacteria
					Root vegetable	Tomato		Total endophytic bacteria
						Carrot		*Actinomycetes*
						Onion		*Pseudomonas spp.*
								*E. coli*
								*Staphylococcus* spp.
								*Salmonella* spp.
10	2017	India	Market	N/A	Fruit	Cucumber	60	Aerobic mesophilic count
					Root vegetable	Tomato	60	Psychotropic count
					Leafy vegetable	Radish	60	Coliform count
					Herbs	Carrot	60	Yeast and mold count
						Beetroot	60	*E. coli*
						Cabbage	60	*E. coli* O157:H7
						Spinach	60	*Salmonella* spp.
						Coriander	60	*Listeria monocytogenes*
								*Exiguobacterium* spp.
11	2018	India	Market	N/A	Herbs	Coriander	60	*Salmonella* spp.
12	2018	India	Market Roadside food seller	3 5 1	Fruit	Cantaloupe	17	*E. coli*
					Herbs	Cucumber	18	*Salmonella* spp.
						Pepper	20	Shiga toxin-producing *E. coli*
						English cucumber	15	Enterohemorrhagic *E. coli*
						Mint and Cilantro	15	Total *Bacteroidales*
13	2018	India	Market Farm	70 40	Fruit	Tomato	80	*E. coli*
					Root vegetable	Cantaloupe	60	*Salmonella* spp.
					Leafy vegetable	Cucumber	80	
						Carrot	80	
						Radish	80	
						Spinach	140	
14	2019	Bangladesh	Market	3 3 6	Fruit	Tomato	30	Total aerobic count
					Vegetable	Cucumber	30	Total coliform count
					Herbs	Lettuce	30	*E. coli*
						Coriander	30	*Salmonella*
								*Staphylococcus* spp.
15	2019	Bangladesh	Farm	4	Fruit	Tomato	40	Total viable count
					Leafy vegetable	Green chilli		Total *E. coli* count
					Environment	Malabar spinach		Total *Salmonella* count
						Red spinach		*E. coli*
								*Salmonella* spp.
						Manure	60	*Salmonella* spp.
						Water	20	*E. coli*
								*Salmonella* spp.
						Soil	40	*E. coli*
								*Salmonella* spp.
16	2019	Nepal	Restaurant Hotel	N/A	Fruit	Cucumber	72	*E. coli*
					Root vegetable	Carrot	72	*Salmonella* spp.
					(Ready-to-eat salad)	Radish	72	
17	2019	India	Market Household	14 8	Vegetable		63	*E. coli*
								*Klebsiella* spp.
								*Enterobacter*
								*Citrobacter*
18	2019	Pakistan	Market	N/A	Root vegetable	Carrot	12	*Staphylococcus* spp.
					Vegetable	Turnip	12	*Bacillus* spp.
						Cabbage	12	*Citrobacter* spp.
								*Enterobacter* spp.
								*Exiguobacterium* spp.
								*Lysinibacillus fusiforms*
								*Arthobacter nicotianae*
								*Burkholderia cepacia*
								*Acinetobacter* spp.
								*Stenotrophomonas maltophilia*
								*Serratia* spp.
								*Pantoea dispersa*
								*Khyvera cryecrescens*
								*Klebsiella pneumoniae*
								*Pseudomonas putida*
19	2020	India	Market	N/A	Fruit	Grapes	1	*E. coli*
					Root vegetable	Pear	2	*Klebsiella* spp.
					Vegetable	Apple	4	*Enterobacter* spp.
						Chilli pepper	23	*Proteus* spp.
						Tomato	28	*Citrobacter* spp.
						Cucumber	28	*Providencia* spp.
						Onion	30	*Acinetobacter* spp.
						Ginger	14	*Pseudomonas* spp.
						Carrot	11	
						Garlic	2	
						Radish	2	
						Cabbage	5	
20	2021	India	Farm	3 1	Environment	Soil	4	Total bacterial count
								Proteobacteria
								Acidobacteria
								Actinobacteria
								Chloroflexi
21	2021	India	Farm	18 32	Environment	Manure	50	
22	2021	India	Market	14 25 9 7	Leafy vegetable	Spinach	220	*E. coli*
					Vegetable	Fenugreek	120	*Salmonella* spp.
						Cabbage	90	
					Herbs	Peppermint	170	
						Coriander	230	

The grey color means the particular study doesn’t report any microorganism. N/A means not applicable.

**Table 5:** ARG reported on fresh produce and agro-ecosystems in the included study article.

Article No.	Country	Antibiotic-Resistant Genes Tested	*E. coli*				*Salmonella* spp.				Methodology		
			Cow Dung	Soil	Water	Vegetable	Cow Dung	Soil	Water	Vegetable	Primer Sequence (5′-3′)	Approxband Size (bp)	Annealing Temp. (°C)
15	Bangladesh	*ereA*	43.18% (19/44)	36.36% (12/33)	37.5% (3/8)	30.77% (8/26)	41.02% (16/39)	34.61% (9/26)	0.0% (0/5)	26.67% (4/15)	F: GCCGGTGCTCATGAACTTGAG R: CGACTCTATTCGATCAGAGGC	419	52
		*tetA*	88.89% (40/45)	82.35% (28/34)	75% (6/8)	80.77% (21/26)	84.61% (33/39)	80.00% (20/25)	80.00% (4/5)	75.00% (12/16)	F: GGTTCACTCGAACGACGTCA R: CTGTCCGACAAGTTGCATGA	577	57
		*tetB*	46.67% (21/45)	38.23% (13/34)	37.5% (3/8)	38.46% (5/18)	41.02% (16/39)	36.00% (9/25)	20.00% (1/5)	31.25% (5/16)	F: CCTCAGCTTCTCAACGCGTG R: GCACCTTGCTGATGACTCTT	634	56
		*SHV*	28.57% (10/35)	26.92% (7/26)	0% (0/6)	27.78% (44/96)	24.00% (6/25)	25.00% (4/16)	0.0% (0/2)	16.7% (1/6)	F: TCGCCTGTGTATTATCTCCC R:CGCAGATAAATCACCACAATG	768	52

## Data Availability

The study data are available upon request.

## Supplementary Materials

The following supporting information can be downloaded at: https://www.mdpi.com/article/10.3390/microorganisms12112267/s1, Table S1: Additional information.
